# How robust is the association between school-related stress and internalizing mental health problems? A specification curve analysis

**DOI:** 10.1186/s12888-025-06829-w

**Published:** 2025-04-22

**Authors:** Björn Högberg

**Affiliations:** 1 Department of Social Work, Umeå University, Umeå, SE-901 87 Sweden; 2 Centre for Demographic and Ageing Research, Umeå University, Umeå, SE-901 87 Sweden

**Keywords:** Academic stress, Academic pressure, School, Mental health, Adolescents, Students

## Abstract

**Background:**

A recent review of research on the association between school-related stress and internalizing problems found growing scholarly interest in the topic, but also raised questions concerning the quality and reliability of the existing knowledge base. The aim of this study was to investigate how robust the association between school-related stress and internalizing problems is to differences in model specifications.

**Methods:**

Longitudinal survey data from between 2,991 and 4,845 Swedish adolescent students aged 13–16 years were used. A total of 57,322 different models were estimated, varying the choice of sample, measure of internalizing problems, functional form, statistical method, and combinations of included control variables. The results were summarized using specification curve analysis.

**Results:**

Most estimates of the association between school-related stress and internalizing problems were statistically significant at the 5% level. The choice of sample, outcome, functional form, and control variables had a limited influence on the size and significance of the estimates, but the estimates were markedly smaller and mostly non-significant in models investigating lagged effects.

**Conclusions:**

This study showed that school-related stress is a robust predictor of internalizing problems as long as the association is assumed to be contemporaneous, while evidence for lagged effects was weaker. A key conclusion is that the choice of whether to estimate lagged or contemporaneous effects may be the most consequential in studies on school-related stress and internalizing problems or similar topics.

**Supplementary Information:**

The online version contains supplementary material available at 10.1186/s12888-025-06829-w.

## Background

Rates of internalizing mental health problems have increased among adolescents across high-income countries [1, 2]. Internalizing problems encompass symptoms related to anxiety, fear, sadness, depressed mood, feelings of worthlessness, and social withdrawal. Psychosomatic health complaints such as headaches, stomachaches, or feeling tense can also be considered forms of internalizing problems [3–5]. Known risk factors for internalizing problems include stressful life events and prolonged exposure to stress [6–8]. One of the most commonly reported sources of stress among adolescents is stress or pressure related to school, including stress related to academic demands, achievements, expectations, or school-based social interactions [9–16].

Recently, calls have been made to “take [school-related stress] seriously” as a risk factor for internalizing problems, and as a potential driver of the worrying trends in such problems in adolescents [17, 18]; see also [1, 2, 19]. Accordingly, a recent systematic review of research on the association between school-related stress and internalizing problems found growing scholarly interest in the topic, with 40 of the 52 included studies having been published since 2010, and 20 since 2020 [20]. However, although 48 of the 52 studies reported positive associations between school-related stress and internalizing problems, “most studies were cross-sectional“ and many “insufficiently accounted for [confounders]” [20], prompting calls for more longitudinal research that includes a broader range of confounders.

Responding to this call, the aim of this study was to investigate how robust the association between school-related stress and internalizing problems is to differences in model specifications. To this end, longitudinal survey data on Swedish adolescent students aged 13–16 years were analyzed using specification curve analysis [21]. Specification curve analysis is a statistical technique used to explore the robustness of research results by systematically varying possible model specifications. It allows researchers to assess the extent to which results are dependent on specific, and often arbitrary, choices made during the research process, thus enhancing the transparency of reported findings and reducing the scope for p-hacking or other unsound research practices. In this study, a total of 57,322 different models were estimated, varying the choice of sample, measure of internalizing problems, functional form, statistical method, and control variables.

## Data and methods

Data from the Evaluation Through Follow-up (ETF) survey were used. ETF is a repeated longitudinal survey on different cohorts of Swedish students, conducted by Statistics Sweden and the University of Gothenburg [22, 23]. The 2004 cohort, which is the only cohort with data on both school-related stress and internalizing problems from more than one wave, was included in the analysis. The sampling frame consisted of all students attending grade 3 of compulsory school in Sweden in 2014. From this frame, 9,794 students, or around 10% of the total grade 3 student population in Sweden, were sampled through a stratified cluster-based design. Schools were the primary sampling unit and were sampled with probability proportional to their number of students and within strata defined by school ownership. All students in the sampled schools were invited to respond to the survey. Students and/or their guardians had the right to decline participation.

Students were followed longitudinally in grades 6 (year 2017; *n* = 4,845) and 9 (year 2020; *n* = 2,991), when most were 13 and 16 years old, respectively. Response rates were 53% in grade 6 and 26% in grade 9 [24]. Weights calibrated based on information from administrative population registers were used to adjust for differential non-response rates. In supplementary analyses, the 1998 cohort was also used. This cohort had a higher grade 9 response rate (48%) but did not include data on internalizing problems in grade 6.

### Dependent variable: internalizing problems

Students were asked how often during the last six months they experienced specific internalizing problems. Using the same data, Giota and Gustafsson [25] showed that a two-factor solution fitted the data best, separating what they termed “emotional” and “psychosomatic” problems. Emotional problems included feeling sad, feeling irritated or moody, feeling nervous, feeling low, difficulties concentrating, conflicts with peers, and withdrawal from peers. Psychosomatic problems included headaches, stomachaches, feeling tense, feeling giddy, difficulties sleeping, and poor appetite. Response alternatives were Never (1), Rarely (2), Sometimes (3), Often (4), and Always (5). Students’ average scores on the items were used as measures of emotional and psychosomatic problems, respectively, with higher values representing more problems. Cronbach’s alpha for both scales was 0.83 in grade 6 and 0.84 in grade 9. In supplementary analyses, the scales were transformed into categorical variables, with the 20% of students with the highest scores coded 1 and the remaining 80% coded 0.

### Dependent variable: school-related stress

One indicator measuring school-related stress was available in ETF. Students were asked how often they feel stressed in school, with response alternatives ranging from Never (1) to Always (5). The reliability of a single item is difficult to ascertain. However, a similar indicator has been used in the Health Behaviour in School-Aged Children Survey [26]. Moreover, Lindholm et al. [27] reported that a similar global item (“I feel stressed”), though not specifically related to school, was strongly related to more comprehensive and specific measures of stress in Swedish students. In supplementary analyses, the indicator was transformed into a categorical variable, with the response alternative “Always” coded 1 and the remaining alternatives coded 0.

### Control variables

The goal of specification curve analysis is to investigate how sensitive estimates are to variations in reasonable analytical choices that researchers can make. One of the most consequential choices in observational research concerns the choice of control variables. It is well known that researchers using observational data ought to control for confounders, but not for mediators or colliders [28]. Some variables, such as basic socio-demographic characteristics that cannot be caused by either school-related stress or internalizing problems, are clearly confounders. All models therefore adjusted for biological sex (0 = boy; 1 = girl), parental education (five categories, from pre-secondary education to at least four years of university) and migration background (1 = born outside Sweden; 0 otherwise).

The status of most other variables was less clear-cut, with theory and previous research not offering unambiguous advice on which variables should be regarded as confounders, mediators, or colliders. For instance, it is plausible that a heavy workload in the form of homework causes both stress and internalizing problems. If so, time spent on homework should be seen as a confounder and thus be controlled for. However, stressed students may also spend more time on homework to cope with high demands, which in turn could result in internalizing problems if they do not get enough time for rest and recovery. In that case, homework is a mediator and should not be controlled for. It is also possible that both stress and internalizing problems independently affect time spent on homework: doing homework may be a way of coping with high demands, and certain internalizing problems related to depression, such as fatigue or difficulties concentrating, may reduce students’ work capacity and force them to spend more time on homework to keep up. If so, homework is a collider and should not be controlled for.

To examine how sensitive the results are to either inappropriate exclusion of confounders, or inappropriate inclusion of mediators or colliders, all unique combinations of the control variables (except for the socio-demographic characteristics) were included in the analysis. However, the possible combinations increase exponentially with the number of control variables. For instance, six variables yield 63 possible combinations, while 16 variables yield 65,535 combinations. To balance the need for a flexible analysis of the importance of different covariate combinations with a manageable number of models, 12 control variables in total were chosen, yielding 4,095 combinations. In some models, only 11 were included since one variable (student’s grade point average) was only available in grade 9. Besides this variable, priority was given to control variables that were measured in both grades 6 and 9 and thus relevant for longitudinal models.

*Achievement* was measured by students’ final grade point average, representing the average grade in all the 16 or 17 subjects taken in Swedish compulsory school. This was only available for students in grade 9.

Self-reported *schoolwork difficulties* were measured using students’ average score on three items of the type “I find it difficult to keep up in lessons”, with response alternatives ranging from Never (1) to Always (5). Cronbach’s alpha for the scale was 0.67 (grade 6) and 0.69 (grade 9).

*Academic self-concept* was measured using students’ average scores on two sets of items. The first set included items related to general self-concept, such as “How good do you think you are in Mathematics?” with response alternatives ranging from Very poor (1) to Very good (5). The second set included items related to specific tasks, such as how well students can “Read and understand a text [in Swedish]”, with response alternatives ranging from Very poorly (1) to Very well (5). Cronbach’s alpha was 0.42 (grade 6) and 0.48 (grade 9) for the general self-concept scale and 0.69 (grade 6) and 0.67 (grade 9) for the specific self-concept scale. The general self-concept scale was kept in the analysis despite low reliability since including each item separately would increase the number of models to be estimated exponentially.

*Achievement goal orientation* was measured using students’ average scores on two sets of items. The first set included four items related to extrinsic and future-oriented goals, such as “[I] learn so that I can get a well-paid job”. The second set included three items related to relative performance and competitiveness, such as “[I] try to be better than other pupils in the class”. Response alternatives ranged from Never (1) to Always (5). Cronbach’s alpha was 0.88 (grade 6) and 0.79 (grade 9) for the extrinsic goals scale and 0.72 (both grades) for the competitive goals scale.

*Relationships with peers* and *relationships with teachers* were measured using single items: “I feel excluded in school” and “My teachers treat me well in school”, respectively, with response alternatives ranging from Never (1) to Always (5).

*School contentment* or satisfaction was measured using students’ average scores on five items such as “How content are you in your current class?”, with response alternatives ranging from Very content (1) to Not at all content (5). Cronbach’s alpha for the scale was 0.85 (grade 6) and 0.84 (grade 9).

Time spent on homework was measured using a single item: “How much time do you spend on average each week doing home assignments or homework?”, with response alternatives ranging from I don’t do homework (1) to 7 h or more (6) in grade 6 and from No time at all (1) to 4 h or more (5) in grade 9. The two highest response alternatives in the grade 6 item were collapsed into one to correspond with the grade 9 response alternatives.

Two indicators of non-school-related leisure activities were used. *Physical activity* was measured by a single item: “How much time do you spend on average per week doing sports or exercising?”. *Screen time* was measured using students’ average score on two items: ”How much time do you spend on average per week watching TV, movies or YouTube?” and ”How much time do you spend on average per week playing video or computer games?”. Response alternatives ranged from 0 h (1) to ≥ 41 h (6). No data on social media usage were available.

All included variables have been shown to be related either to school-related stress or internalizing problems in adolescents: achievement [29], schoolwork difficulties [25, 30], academic self-concept [31], goal orientations [32, 33], school contentment [34], homework [35], physical activity [36], and screen time [37, 38]. Further information on the measurement of the variables, the construction of scales, as well as summary statistics, is reported in Additional file A.

### Specification curve analysis

Table 1 displays decisions a researcher analyzing the association between school-related stress and internalizing problems using observational data must make, as well as the choices made in the present study. A first decision concerns which data to use. ETF can be analyzed using either cross-sectional or longitudinal data. In this study, both types were used so as to maximize the variance in possible analytical choices. This also reflects the existing literature on the topic, which includes both cross-sectional and longitudinal designs [20]. The cross-sectional analyses used data from both grades 6 and 9, but separately, while the longitudinal analyses included both. A second decision concerns the measure of the outcome. Internalizing problems may be viewed as either dimensional or categorical. In this study, a dimensional approach was used in the main analysis and a categorical approach in supplementary analyses. A third decision concerns the choice of control variables, as discussed in the previous subsection. All socio-demographic control variables, and all possible combinations of the remaining control variables, were included.

**Table 1:** Possible analytical decisions in analyses of ETF data

Decisions to be made	Choices considered in the present study
What sample or data to include: 6th, 9th grade, or both? Cross-sectional or longitudinal data?	All combinations of grades 6 and 9: cross-sectional within both grades and longitudinal across grades.
Which outcomes to include: Emotional and/or psychosomatic problems? Dimensional or categorical measurement?	Both emotional and psychosomatic problems. Dimensional measure in the main analysis, categorical in supplementary analyses.
Which control variables to adjust for: Which variables should be included, and how should they be combined?	12 control variables in total, based on previous research. All possible combinations of these variables: 12 in grade 9, 11 in grade 6 and in longitudinal analyses.
Which statistical model to estimate: Functional form of the association: Linear or non-linear? How to adjust for control variables: Regression vs. matching or balancing? How to model associations over time: Type of longitudinal model?	Linear models in the main analysis, logistic models in supplementary analyses. Regression-based adjustment for control variables. Fixed effects, lagged dependent variable and prospective cohort models in longitudinal analyses.

A fourth decision concerns the choice of statistical model, which in turn incorporates the choice of how to model the functional form of the association, how to adjust for control variables, and how to model the associations over time in the longitudinal data. As for the functional form, the analyses presented in the paper assumed linear associations, while non-linear associations were modeled using logistic regression in supplementary analyses. The results from logistic models were presented separately in Additional file B since odds ratios cannot be directly compared with linear regression coefficients. As for how to adjust for control variables, the main alternatives are regression-based approaches– in which the associations between the control variables, exposure and outcome are modeled simultaneously– and matching or balancing approaches that model the associations between control variables and the exposure before estimating the association between the exposure and the outcome (e.g., propensity score matching). Matching or balancing typically assumes that the exposure is binary, which is not the case with school-related stress. Only regression-based adjustment was therefore used in this study. As for how to model associations over time, dozens of alternative models are available, many of which, however, were not compatible with the data at hand. In this study, three common types of longitudinal models were estimated: fixed effects (FE) models, lagged dependent variable (LDV) models, and prospective cohort models.

FE and LDV models constitute two different ways of adjusting for confounding in longitudinal analysis, and are both popular in social science [39]. FE models only use variation within individuals over time to estimate associations. With two waves of data, FE models are equivalent to first difference models: the change in the outcome between the first and second wave is regressed on the corresponding change in the exposure and control variables. This effectively controls for all time-invariant confounding with constant effects over time. FE models, however, tend to perform poorly in the context of lagged effects of the exposure on the outcome, or lagged effects of the outcome on the exposure (i.e., reverse causation) [39–41]. LDV models adjust for lagged scores of the outcome when estimating the contemporaneous association between the exposure and the outcome, with the rationale that the lagged outcome can adjust for the most important sources of confounding. Estimates from LDV models are more robust to reverse causality compared to FE models [39]. Angrist and Pischke [42] show that FE and LDV models have a useful bracketing property: when FE models are correct, LDV models will underestimate effects, while when LDV models are correct, FE models will overestimate effects.

The prospective cohort model is more popular in epidemiology and public health research. The focal coefficient of these models is the effect of the exposure measured at baseline on the outcome measured at follow-up, adjusted for the outcome measured at baseline. Like the LDV model, but unlike the FE model, the prospective cohort model uses lagged scores on the outcome to adjust for confounding. Unlike both FE and LDV models, the focus of the prospective cohort model is on the lagged rather than contemporaneous effect of the exposure on the outcome.

In sum, the analyses considered linear cross-sectional regression models, separately for grade 6 and 9 students, as well as linear longitudinal models (FE, LDV and prospective cohort models). All models used both emotional and psychosomatic problems as the outcome and included all socio-demographic control variables and all possible combinations of the 11–12 remaining control variables, yielding a total of 24,566 models in the main analyses. In supplementary analyses, another 32,756 models were estimated, yielding a total of 57,322 models. The specification curves were created using the *speccurve* program in Stata [43]. Additional technical details on the models are provided in Additional file A.

## Results

**Fig. 1:**
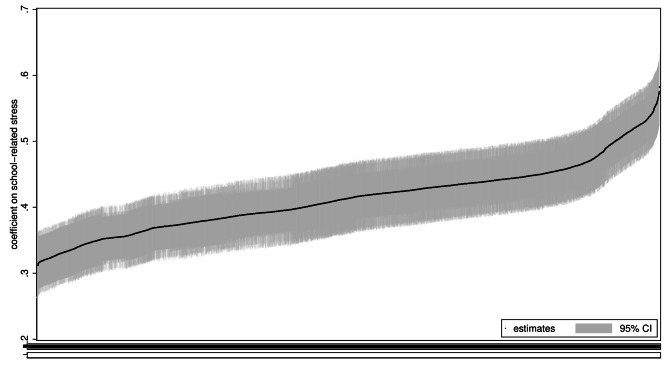
Specification curve analysis of cross-sectional linear regression models. All estimates. *Note*: CI = confidence interval

**Fig. 2:**
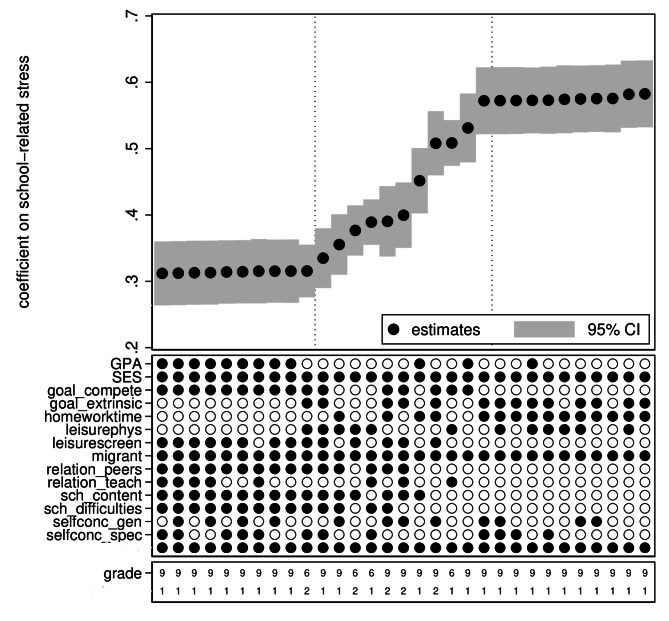
Specification curve analysis of cross-sectional linear regression models. Subset of estimates. *Note*: Outcome: 1 = emotional problems; 2 = psychosomatic problems. CI = confidence interval

Figure 1 shows results of the cross-sectional analyses. Both school-related stress and internalizing problems were standardized (mean = 0; standard deviation = 1) to facilitate comparison of effect sizes across models. The estimates ranged from a low of 0.31 to a high of 0.58, with around 90% of the estimates falling between 0.35 and 0.45. All estimates were significant at the 5% level, as can be seen from the 95% confidence intervals. The distribution of estimates was smooth, and there was no indication that a particular choice of specification resulted in a discrete jump in the effect sizes. The large number of estimates makes it impossible to see which models generate which estimates. Figure 2 therefore shows a subset of the estimates: the 10 largest and smallest estimates, and 10 random estimates in between. The smallest estimates consistently include competitive achievement goals, relationships with peers (i.e., feeling excluded), school contentment, and schoolwork difficulties, while the largest estimates consistently exclude these variables. Likewise, the largest estimates mostly include extrinsic (future-oriented) achievement goals, homework, and leisure-based physical activity, while the smallest estimates mostly exclude these variables.

**Fig. 3:**
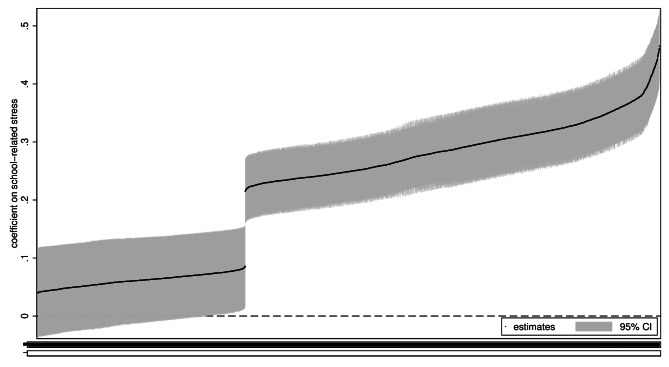
Specification curve analysis of longitudinal linear regression models. All estimates. *Note*: CI = confidence interval

**Fig. 4:**
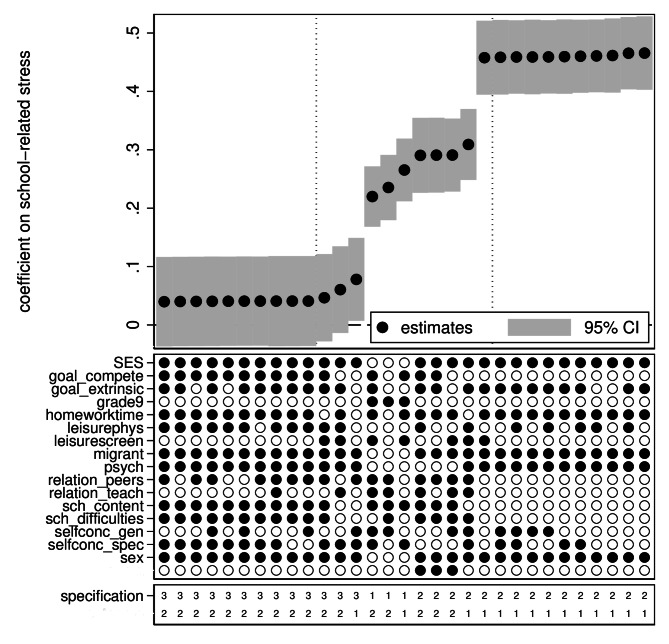
Specification curve analysis of longitudinal linear regression models. Subset of estimates. *Note*: Outcome: 1 = emotional problems; 2 = psychosomatic problems. Specification: 1 = FE model; 2 = LDV model; 3 = prospective cohort model. CI = confidence interval

Figures 3 and 4 show results of the longitudinal analyses. Unlike in Fig. 1, with the corresponding cross-sectional results, there is a discrete break in Fig. 3. The break reflects the markedly smaller estimates from the prospective cohort models compared to the estimates from the FE and LDV models. The estimates from the prospective cohort models ranged from 0.04 to 0.09, and most were not significant at the 5% level. Figure 4 also makes clear that the smallest estimates were with psychosomatic problems as the outcome. Estimates from the FE and LDV models were more smoothly distributed and also overlapped with each other to a considerable extent. Overall, estimates from the FE models were smaller, with the smallest being 0.22, and estimates from the LDV models larger, with the largest being 0.45.

### Supplementary analyses

Additional file B shows that the results were qualitatively similar when categorical transformations of the exposure and outcome, and logistic regression models, were used. Additional file C shows that the cross-sectional results for grade 9 students were very similar when the 1998 cohort, with higher response rates, was used. Additional file D shows that stress or internalizing problems in grade 6 did not predict non-response in grade 9.

## Discussion

Following calls for more longitudinal studies that include a broad range of confounders in research on the association between school-related stress and internalizing problems [18, 20], the aim of this study was to investigate how robust the association between school-related stress and internalizing problems is to differences in model specifications. To this end, specification curve analysis [21] was used to analyze longitudinal survey data on Swedish adolescent students aged 13–16, varying the choice of sample, measure of internalizing problems, functional form, statistical method, and combinations of included control variables. A total of 57,322 different regression models were estimated.

Two main results ought to be highlighted. First, the association between school-related stress and internalizing problems was largely robust, with around 90% of all estimates being significant at the 5% level. This is in line with the review by Steare et al. [20], which found that 48 of 52 included studies reported at least one positive association between school-related stress and internalizing problems. Most of the standardized coefficients in the present study ranged between 0.25 and 0.45, which is similar to estimates reported in, for instance, Kristensen et al. [13]. Since the indicator of school-related stress could only take on five different values, this means that going from the lowest to the highest level of stress was typically associated with between a 1.25 and 2.25 standard deviation increase in internalizing problems.

However, and second, the longitudinal estimates were generally somewhat smaller than the cross-sectional ones. Longitudinal data can more effectively adjust for confounding and are therefore, despite entailing other potential sources of bias (e.g., due to attrition), generally regarded as superior from the perspective of causal inference. The most marked difference was between the prospective cohort models and all other specifications. The prospective cohort model is identical to the LDV model, apart from when the focal exposure (school-related stress) is measured: contemporaneously in the LDV model or at baseline in the prospective cohort model. Thus, the results from the different specifications can be summarized as follows: the choice of outcome, including whether the outcome is operationalized as dimensional or categorical, seems to be least consequential for the results. The choice of which variables to include and whether to use cross-sectional or longitudinal data has some bearing on the results, but not a particularly large one as long as the association between school-related stress and internalizing problems is measured contemporaneously or as change-scores. The by far most consequential choice concerns whether to estimate lagged effects of school-related stress.

The choice between contemporaneous and lagged effects must be made on substantive grounds, using theory, reason, and relevant previous research [44]. On the one hand, causality operates in time and strictly contemporaneous or instantaneous effects, down to the most miniscule nanosecond, are impossible. On the other hand, the gaps between measurement points in a given data set may not match the lags operating in the real world [39, 41, 44]. What are, then, the temporal properties of the underlying causal process through which school-related stress is assumed to affect internalizing problems? Extant theory and research are arguably not sufficiently developed to allow for a precise answer concerning the appropriate lags in the context of school-related stress and internalizing problems. This context is particularly complex since it concerns associations between mental and emotional phenomena that are best understood as dimensional. Thus, there are no clear points that mark when the exposure (school-related stress) first occurred and thus started to exert its influence. However, it seems plausible that both almost immediate and more long-term effects are relevant and that they do not need to be mutually exclusive.

Some internalizing problems included in this study– such as irritation, nervousness, tenseness, giddiness, or sleep problems– may be almost instantaneously affected by stress. They are also conceptualized as manifestations of school-related stress by students themselves [45]. Thus, even though strictly contemporaneous effects are, as stated, impossible, the temporal dynamics may nevertheless be well approximated as such [39, 41, 44]. Long-term lagged effects may occur due to stress sensitization [46], whereby earlier exposure to school-related stress increases vulnerability to later stressors, including stressors not related to school. Thus, there may be a heightened risk of internalizing problems at later time points also in the absence of school-related stress at that particular time point. A special form of long-term effect is the adverse effects of chronic stress [47], with the gradual “wear and tear of the body” resulting from chronic stress making the individual progressively more susceptible to developing internalizing problems over time. Both stress sensitization and chronic stress are processes that may unfold over several years, thus suggesting that extended lag-times may be relevant.

Nonetheless, the finding that the longitudinal associations were smaller than the cross-sectional ones suggests that the cross-sectional associations are biased upwards due to unmeasured confounding. Thus, the general proposition that longitudinal data are preferable from the perspective of causal inference is borne out in the context of school-related stress and internalizing problems. Moreover, the fact that the prospective cohort model yielded small and mostly non-significant estimates suggests that school-related stress does not cause persistent harm once removed. One interpretation of this is that students are adaptive, which, in turn, suggests that interventions to reduce stress can yield fairly swift mental health benefits. This also suggests that information on school-related stress may have limited prognostic value for practitioners in predicting future internalizing problems, at least over and above information on current internalizing problems. However, it should be noted that this conclusion is partly contradicted by previous prospective cohort studies on the topic, all of which found evidence of lagged and long-term effects. In these studies, time from baseline measurements to follow-up varied from 6 to 12 months [48], to one year [49] and five years [50], as compared to three years in the present study.

### Limitations

One major limitation of the present study was the low response rates, especially in grade 9. Response rates in ETF have declined in recent decades. The causes of this decline– which is part of a broader trend– are not known, but may include lower trust in institutions and authorities, stricter privacy concerns, or time limitations. In the case of ETF, the lower response rate in grade 9 compared to grade 6 may be because the grade 6 survey was conducted in school whereas the grade 9 survey was sent to the students’ homes. Another reason may be that participation in the grade 9 survey required written informed consent from the students. Of the 7,048 students that did not provide data in grade 9, 964 (14%) completed the survey but did not fill out the consent form [24]. Moreover, the response rate in grade 9, surveyed in the spring of 2020, may have been influenced by the COVID-19 pandemic.

Low response rates can undermine the external validity as well as the reliability of results due to possible non-response bias. It is difficult to ascertain the direction of any non-response bias in this case. In general, the magnitude of the associations will be biased upward if students with high levels of both school-related stress and internalizing problems were more likely to participate than students with high levels of only one of these measures. The finding that the results were very similar in samples with higher response rates– grade 6 and grade 9 in the 1998 cohort– suggests that at least the cross-sectional results may be robust to potential non-response bias. Moreover, supplementary analyses showed that stress and internalizing problems in grade 6 did not, either independently or in combination, predict non-response in grade 9 (Additional file D). The possibility that non-participation in both surveys was related to stress or internalizing problems cannot, however, be ruled out. It should also be noted that the use of calibration weights corrected for higher non-response among boys, low-achieving students, and students with low socioeconomic backgrounds [24].

A second limitation is that ETF only contains a single indicator of school-related stress. This item may have limited reliability and validity compared to more comprehensive scales. The use of a single item also means that specific subsets of school-related stress identified in previous research– such as stress related to achievements, school-leisure conflicts, or social interactions in school [9–11, 45]– could not be identified. A third limitation is that only two waves of data were used in the longitudinal analyses. More, and more proximally spaced, measurement points would enable more advanced and credible analyses of reciprocal and lagged effects. A fourth limitation is that both school-related stress and internalizing problems were self-reported, making the results sensitive to common methods bias [51].

Moreover, the specification choices considered in this study do not encompass all possible choices that can be made using ETF data, let al.one other sources of data. For instance, it is possible that confounding factors interact in complex, non-linear ways that are not captured by the linear and additive effects assumed in linear regression models. Estimating all second- or higher-order interactions of all possible control variable combinations would, however, be unwieldy. Likewise, the data were not compatible with some state-of-the-art methods for longitudinal analysis. For instance, event study and synthetic control designs, popular in the social sciences [52], are most suitable in contexts where the exposure is a discrete event. Likewise, random-intercept cross-lagged panel models designed to simultaneously handle unobserved time-invariant confounding and lagged effects [53, 54], and that are popular in psychology [13], require more than two waves of data.

## Conclusion

Based on a specification curve analysis of 57,322 regression models, this study showed that school-related stress is a robust predictor of internalizing problems, regardless of the choice of data and sample, measurement of the outcome, assumed functional form, included control variables, or statistical model. The one exception was the prospective cohort models that modeled lagged effects adjusted for baseline values of the outcome, which generally found these effects to be small and non-significant. A key takeaway is therefore that the choice of whether to estimate lagged or contemporaneous effects may be the most consequential one in studies on school-related stress and internalizing problems or similar topics. Research using multi-wave data or high-frequency longitudinal designs can make further headway in understanding the temporal dynamics underlying the association between school-related stress and internalizing problems. Such research would, moreover, gain from incorporating the flexibility and transparency inherent in specification curve analysis.

## Electronic supplementary material

Below is the link to the electronic supplementary material.

Supplementary Material 1

## Data Availability

Data are not available for public use. Please see the ETF website for more information: https://www.gu.se/utvardering-genom-uppfoljning.

## Abbreviations

ETF: Evaluation through follow-up
FE: Fixed effects
LDV: Lagged dependent variable
