# Supplementary material for: How robust is the association between school-related stress and internalizing mental health problems? A specification curve analysis
Source: BMC Psychiatry. 2025 Apr 22;25:413. doi: 10.1186/s12888-025-06829-w (PMC12016417; doi:10.1186/s12888-025-06829-w)
Supplement: Supplementary file 1 — Supplementary Material 1 [file 12888_2025_6829_MOESM1_ESM.docx]

**Supplementary materials for *How robust is the association between school-related stress and internalizing problems? A specification curve analysis***

**Additional file** **A. Summary statistics, measurement of variables, and model specifications**

**Additional file B. Results of logistic regression models**

**Additional file C. Cross-sectional results for the 1998 cohort**

**Additional file D. Attrition analyses**

**Additional file A. Summary statistics, measurement of variables, and model specifications**

**Table A1. Summary statistics and measurement of all variables**

| **Indicator** | **Measurement** | **Mean, standard deviation (SD), and range** |
| --- | --- | --- |
| *Outcomes* |  |  |
| Emotional problems | During the last six months, have you…  Experienced difficulties concentrating  Felt sad  Felt irritated or moody  Felt nervous  Felt low  Experienced conflicts with peers  Withdrawn from peers  Response alternatives: Always (5), Often (4), Sometimes (3), Rarely (2), Never (1).  The indicator shows students’ average score on the included items. The indicator was standardized in the analysis, with mean=0 and standard deviation=1. | Mean=2.66  SD=0.83  Range=1-5 |
| Psychosomatic problems | During the last six months, have you…  Experienced difficulties sleeping  Had headaches  Had stomach-aches  Felt tense  Felt giddy  Had poor appetite  Response alternatives: Always (5), Often (4), Sometimes (3), Rarely (2), Never (1).  The indicator shows students’ average score on the included items. The indicator was standardized in the analysis, with mean=0 and standard deviation=1. | Mean=2.44  SD=0.85  Range=1-5 |
| *Exopsure* |  |  |
| School-related stress | How do the following statements describe your situation in school?  I feel stressed  Response alternatives in grade 6: Always (5), Often (4), Sometimes (3), Rarely (2) and Never (1).  Response alternatives in grade 9: Always / almost always (5), Often (4), Sometimes (3), Rarely (2) and Never / almost never (1).  The indicator was standardized in the analysis, with mean=0 and standard deviation=1. | Mean=3.05  SD=1.23  Range=1-5 |
| *Sociodemographic control variables* |  |  |
| Sex | Legal sex  Boy=0; girl=1 | Mean=0.49  SD=0.50  Range=0-1 |
| Socioeconomic status (SES): Parental education | Highest attained education of either biological parent. Pre-secondary education (1)  Secondary education (2)  Post-secondary education < 2 years (3)  Higher education 2 or 3 years (4)  Higher education >=4 years (5) | Mean=3-36  SD=1-22  Range=1-5 |
| Immigrant background | Country of birth.  0=Sweden; 1=Outside Sweden | Mean=0.05  SD=0.23  Range=0-1 |
| *Other control variables* |  |  |
| Achievement: grade point average | The sum of the best 16 subject grades in the 9^th^ grade of compulsory school. The highest grade (A) gives 20 points.  A grade below pass-level (F) gives 0 points. | Mean=225.02  SD=65.72  Range=0-320 |
| Schoolwork difficulties | How do the following statements describe your situation in school?  I find it difficult to keep up in lessons  I easily give up if I get a difficult task to do in school  I need more help than what I get from my teachers  Response alternatives in grade 6: Always (5), Often (4), Sometimes (3), Rarely (2) and Never (1).  Response alternatives in grade 9: Always / almost always (5), Often (4), Sometimes (3), Rarely (2) and Never / almost never (1).  The indicator shows students’ average score on the included items. | Mean=2.39  SD=0.96  Range=1-5 |
| General academic self-concept | How good do you think you are in …  Swedish  English  Mathematics  Response alternatives: Very good (5), Quite good (4), Neither good nor poor (3), Quite poor (2), Very poor (1)  The indicator shows students’ average score on the included items. | Mean=3.81  SD=0.68  Range=1-5 |
| Specific academic self-concept | How well do you think you are able to accomplish the following in [Swedish / English / Mathematics]?  Read and understand a text [in Swedish]  Write a story [in Swedish]  Read and understand a text [in English]  Mental arithmetic/estimation [in Mathematics]  Solve mathematical problems [in Mathematics]  Solve equations [in Mathematics]  Response alternatives: Very good (5), Quite good (4), Neither good nor poor (3), Quite poor (2), Very poor (1)  The indicator shows students’ average score on the included items. | Mean=4.00  SD=0.64  Range=1-5 |
| Achievement goal orientation: extrinsic and future-oriented goals | How often do you try and do the following things in school?  Learn so that I can get a well-paid job  Learn so as to be able to look after myself when I am an adult  Learn so that I can get a job that I like  Be successful in school so that I can get a place on a good educational program  Response alternatives: Always (5), Almost always (4), Sometimes (3), Almost never (2), Never (1).  The indicator shows students’ average score on the included items. | Mean=4.19  SD=0.82  Range=1-5 |
| Achievement goal orientation: competitive and performance-oriented goals | How often do you try and do the following things in school?  To be better than other pupils in the class  To show my teacher that I am smarter than other pupils  Learn so that other people will think I am clever?  Response alternatives: Always (5), Almost always (4), Sometimes (3), Almost never (2), Never (1).  The indicator shows students’ average score on the included items. | Mean=2.70  SD=1.04  Range=1-5 |
| Relationships with peers | How do the following statements describe your situation in school?  I feel excluded in school  Response alternatives in grade 6: Always (1), Often (2), Sometimes (3), Rarely (4) and Never (5).  Response alternatives in grade 9: Always / almost always (1), Often (2), Sometimes (3), Rarely (4) and Never / almost never (5).  The indicator shows students’ average score on the included items. | Mean=4.23  SD=1.06  Range=1-5 |
| Relationships with teachers | How do the following statements describe your situation in school?  Item wording in grade 6: My teachers treat me well  Item wording in grade 9: I Feel unfairly treated by teachers  Response alternatives in grade 6: Always (5), Often (4), Sometimes (3), Rarely (2) and Never (1).  Response alternatives in grade 9: Always / almost always (1), Often (2), Sometimes (3), Rarely (4) and Never / almost never (5). | Mean=4.26  SD=0.97  Range=1-5 |
| School contentment | How content are you…  In your current school?  In your current class?  With the teachers?  With the school work?  With other pupils?  Response alternatives: Very content (5), Somewhat content (4), Neither content nor discontent (3), Somewhat discontent (2), Very discontent (1)  The indicator shows students’ average score on the included items. | Mean=4.11  SD=0.72  Range=1-5 |
| Homework | How much time do you spend on average each week doing home assignments or homework?  Response alternatives in 6^th^ grade: 7 hours or more (6), 5-6 hours (5), 3-4 hours (4), 1-2 hours (3), Less than 1 hour (2), Do not do homework (1).  Response alternatives in 9^th^ grade: Four hours or more (5), 3-4 hours (4), 1-2 hours (3), Less than an hour (2), No time at all (1) | Mean=3.48  SD=1.19  Range=1-6 |
| Leisure: screen time | How much time do you spend on average per week on the following…  Watch TV, movies or YouTube  Play video games or games on computer  Response alternatives: 0 h/week (1), 1-4 h/week (2), 5-10 h/week (3), 11-20 h/week (4), 21-40 h/week (5), >41 h/week (6)  The indicator shows students’ average score on the included items. | Mean=2.92  SD=1.09  Range=1-6 |
| Leisure: physical activity | How much time do you spend on average per week on the following…  Sports, exercises  Response alternatives: 0 h/week (1), 1-4 h/week (2), 5-10 h/week (3), 11-20 h/week (4), 21-40 h/week (5), >41 h/week (6) | Mean=2.79  SD=1.11  Range=1-6 |

Note that, while the wording of the questions and items are identical in grades 6 and 9 for all variables save for relationship with teachers, the response alternatives differ in some cases. This is unfortunate and may add unnecessary noise to the estimation of the effects of the covariates in the fixed effects models that rely on variation in variables within individuals over time for estimation.

**Model specifications**

Cross-sectional, linear (ordinary least squares) regression model:

$Y_{i}={\beta_{0}+\beta_{1}S}_{i}+{\beta_{2}X}_{i}+\varepsilon_{i}$ (Equation 1)

Where the i subscript denotes individual students, Y is the outcome (emotional or psychosomatic problems), S is school-related stress, X is a vector of covariates that varies across models, and ε is an individual error term. The βs denote regression coefficients, with $\beta_{0}$ being the intercept. Equation 1 is fitted separately in grade 6 and grade 9, and separately for emotional and psychosomatic problems.

Fixed effects (FE) model:

$Y_{it}={{\alpha_{i}+\beta}_{0}+\beta_{1}S}_{it}+{\beta_{2}X}_{it}+\beta_{3}G_{i}+\varepsilon_{it}$ (Equation 2)

Where the i subscript denotes individual students and the t subscript denotes time (or measurement points or grades; that is grade 6 and 9). Unlike the cross-sectional model (Equation 1), the fixed effects model include $\alpha_{i}$, which is a unit fixed effect, and $G_{i}$, which is a time (or grade-level) fixed effect. Equation 2 is fitted separately for emotional and psychosomatic problems.

Lagged dependent variable (LDV) model

$Y_{i,t+0}={\beta_{0}+\beta_{1}S}_{i,t+0}+{\beta_{2}X}_{i,t+0}+{\beta_{3}Y}_{i t-1}+\varepsilon_{it}$ (Equation 3)

Where the i subscript denotes individual students and the t subscript denotes time. The outcome, exposure (school-related stress) and the covariates are all measured in time t, that is, grade 9; hence the $t+0$ subscript. Equation 3 also includes $Y_{i t-1}$ as a covariate, which is the one-wave lagged value of the outcome (measured in grade 6). Equation 2 is fitted separately for emotional and psychosomatic problems.

Prospective cohort model

$Y_{it}={\beta_{0}+\beta_{1}S}_{i t-1}+{\beta_{2}X}_{i t-1}+{\beta_{3}Y}_{i t-1}+\varepsilon_{it}$ (Equation 4)

Where the i subscript denotes individual students and the t subscript denotes time. The outcome is measured in time t, that is, grade 9; hence the $t+0$ subscript. The exposure (school-related stress) and the covariates are measured with a one-wave lag, in grade 6. Equation 4 also includes $Y_{i t-1}$ as a covariate, which is the one-wave lagged value of the outcome. Equation 4 is fitted separately for emotional and psychosomatic problems.

**Additional file B. Results of logistic regression models**

Figures B1-B4 report log odds. Positive log odds (greater than 0) indicate a positive association, while negative log odds (smaller than 0) indicate a negative association. Confidence intervals that do not include 0 indicate associations that are significant at the 5% level.

**Fig.B1 Specification curve analysis of cross-sectional logistic regression models. All estimates.**


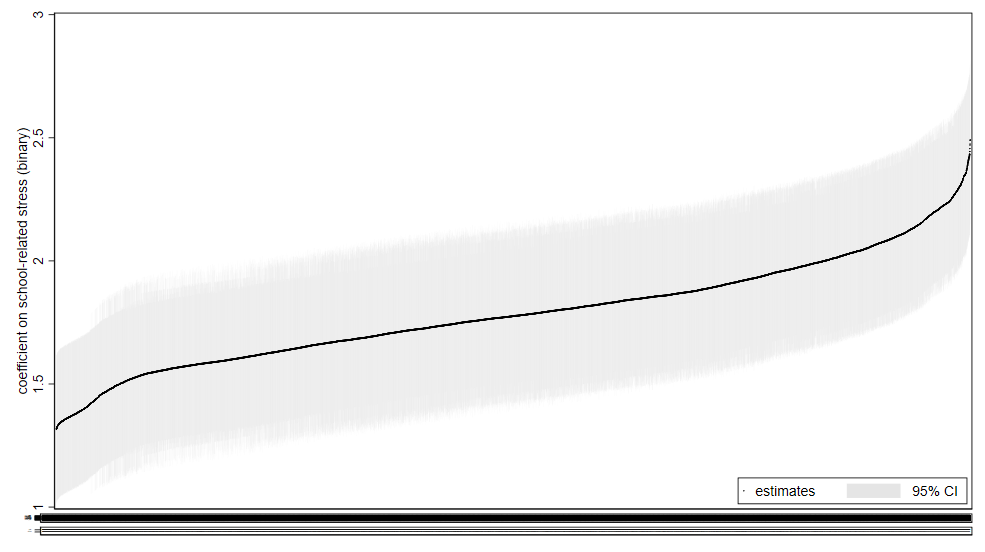
Note: CI = confidence interval.

**Fig.B2 Specification curve analysis of cross-sectional logistic regression models. Subset of estimates.**

Note: Outcome 1 = emotional problems; 2 = psychosomatic problems. CI = confidence interval.

**Fig.B3 Specification curve analysis of longitudinal logistic regression models. All estimates.**


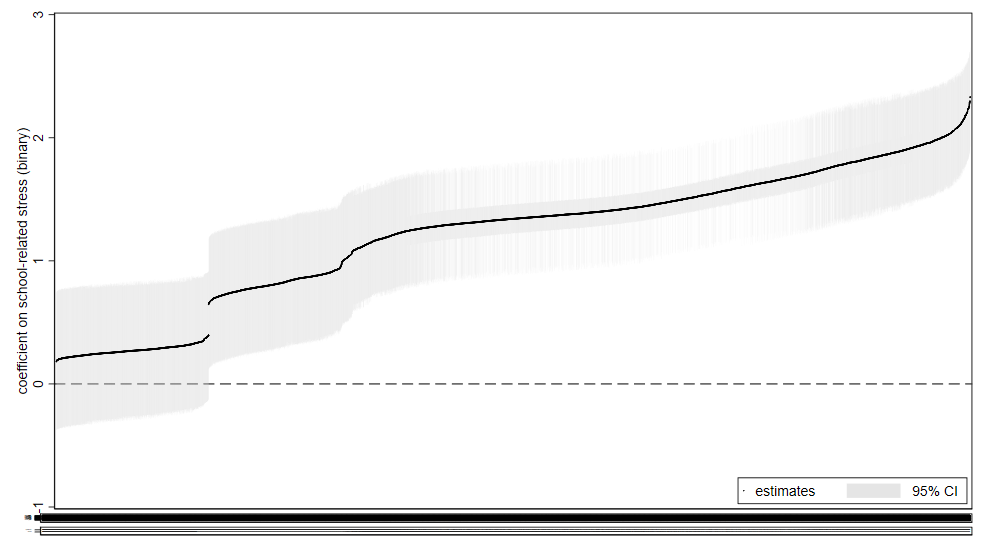
Note: CI = confidence interval.

**Fig.B4 Specification curve analysis of longitudinal logistic regression models. Subset of estimates.**

Note: Outcome: 1 = emotional problems; 2 = psychosomatic problems. Specification: 1 = FE model; 2 = LDV model; 3 = prospective cohort model. CI = confidence interval.

**Additional file C. Cross-sectional results for the 1998 cohort**

**Fig.C1 Specification curve analysis of cross-sectional linear regression models. All estimates.**


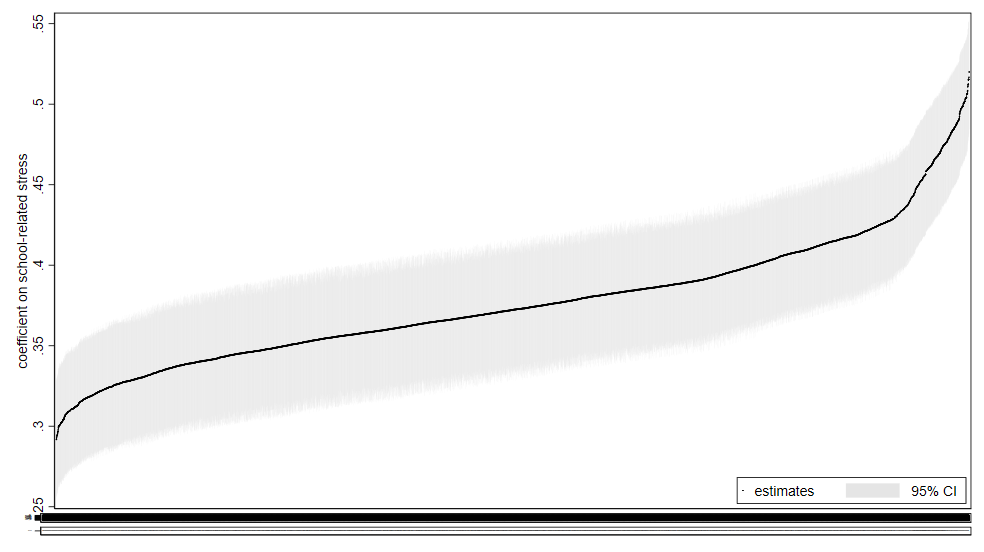
Note: CI = confidence interval.

**Fig.C2 Specification curve analysis of cross-sectional linear regression models. Subset of estimates.**

Note: Outcome 1 = emotional problems; 2 = psychosomatic problems. CI = confidence interval.

**Additional file D. Attrition analyses**

Table D1 shows results from logistic regression models with missing data on school-related stress in grade 9 as the outcome variable. The variable is coded 0 for students with missing data on school-related stress in grade 9, and 1 for students with data on school-related stress in grade 9. The independent variables are school-related stress in grade 6, emotional problems in grade 6, and psychosomatic problems in grade 6. All independent variables have been standardized (M=0; SD=1).

Only data from the 2004 cohort is used since the 1998 cohort has no data on internalizing problems in grade 6.

Coumns 1-3 show bivariate results, with each independent variable entered separately into the models. Columns 4 shows results when school-related stress in grade 6 is interacted with emotional problems in grade 6, and column 5 results when school-related stress in grade 6 is interacted with psychosomatic problems in grade 6. All coefficients are small and non-significant, and there are no indications that non-response in grade 9 is related to school-related stress or internalizing problems in grade 6.

**Table D1. Logistic regression models with non-missing data on school-related stress in grade 9 as the outcome**

|  | Column 1 | Column 2 | Column 3 | Column 4 | Column 5 |
| --- | --- | --- | --- | --- | --- |
| Stress grade 6 | 0.887 |  |  | 0.761 | 0.861 |
|  | [0.526,1.494] |  |  | [0.502,1.155] | [0.530,1.396] |
| Emotional problems grade 6 |  | 0.810 |  | 1.654 |  |
|  |  | [0.414,1.588] |  | [0.859,3.184] |  |
| Psychosomatic problems grade 6 |  |  | 0.732 |  | 1.269 |
|  |  |  | [0.386,1.387] |  | [0.631,2.552] |
| Stress X Emotional problems grade 6 |  |  |  | 0.744 |  |
|  |  |  |  | [0.482,1.148] |  |
| Stress X Psychosomatic problems grade 6 |  |  |  |  | 0.825 |
|  |  |  |  |  | [0.612,1.112] |

Note. Table shows odds ratios, with 95% confidence intervals in brackets. * p<0.05, ** p<0.01, *** p<0.001
